# Fatal Case of Cerebral Affection, in Which the Symptoms Underwent Much Temporary Mitigation from the Formation of an Abscess in the Axilla; and in Which Serous Effusion Was Found in the Brain, and a Tumor in the Cerebellum

**Published:** 1826-07

**Authors:** P. M. Latham

**Affiliations:** St. Bartholomew's Hospital.


### CASES

### OBTAINED FROM PUBLIC INSTITUTIONS, AND OTHER AUTHENTIC SOURCES.

---

### CEREBRAL AFFECTIONS.

*Fatal Case of Cerebral Affection, in which the Symptoms underwent much temporary mitigation from the formation of an Abscess in the Axilla; and in which Serous Effusion was found in the Brain, and a Tumor in the Cerebellum.* Treated by Dr. P. M. Latham, at St. Bartholomew's Hospital.

Sarah Davenport, ætat. thirty-five, was admitted into St. Bartholomew's Hospital, on the 22d of November, 1825. She had suffered pain in the head during eight months, but it was only during a fortnight that it had become so severe as to induce her to seek medical advice. During this period she had been treated by cupping and leeches, without benefit; and, at the time of her admission, she had a seton in the nape of her neck. It was understood that, prior to her present disorder, she had had a syphilitic complaint, for which she had been profusely salivated, and of which there was no discoverable symptom remaining.

There was something very remarkable in the first appearance of the woman: her countenance indicated much apprehension and alarm, and her gait was constrained and trembling; but there was no symptom present which denoted the least degree of paralytic affection: she rather walked like a person who is balancing a burden on the head. She exhibited many strongly-marked hysterical symptoms, which were thought to explain, but which in fact contributed to perplex, the nature of the case.

At first she was treated with purgatives (for her bowels were obstinately costive,) and fetid medicines; but the observation of a few days was enough to remove all belief of her complaint being merely hysterical. Upon more careful examination, it was found that her look of terror and her trembling gait were owing to the continual effort she was making to give steadiness to her head. She soon became permanently confined to bed, and was unable to lift her head from the pillow for the greater part of the day and night. In the mean time, her pulse was so feeble as to forbid bleeding from the arm; and, although the throbbing of the head called for the application of leeches to the temples, and cupping glasses to the nape of the neck, the relief obtained by these remedies was too short and too insignificant to hold out a hope of effectual benefit.

It was thought right to try the effect of salivation; but she so soon lost strength under the use of mercury, that its employment was discontinued before its full constitutional impression was obtained.

After the use of oil of turpentine and of sulphate of quinine, both of which aggravated the complaint, it only remained to resort to those remedies which exercise an immediate influence upon the nervous system. Of these, conium first, and afterwards belladonna, were tried fairly, but without benefit.

It was now the middle of January: all the remedies which had been employed had failed; the severity of her pains had greatly increased; her general health was failing to such a degree, as to threaten her speedy dissolution. But now she complained of pain and tenderness in the left axilla, passing round towards the back. Upon examination, the parts were found tense and tumid. In a day or two, fluctuation was discovered; and, from an incision in the axilla, an enormous quantity of well-formed pus gushed out. The discharge of pus continued during three weeks, and amounted to more than a pint daily: it seemed to be derived from the back, and to pass out from beneath the latissimus dorsi.

During the continuance of this discharge, the pain in the head entirely ceased. The patient rose from her bed, walked about the ward, and described herself as exempt from all ailment. She was peculiarly happy, and exulted in the certainty of her restoration to health.

Perhaps it might not be thought unwarrantable to conclude, at this period of the case, that the symptoms referrible to the head, which were so entirely removed, could not depend upon permanent organic disease.

But the purulent discharge began to abate, and at length it ceased altogether; and now the patient no longer remained exempt from complaint. Her former pains returned, and threatened, by their gradual increase, to reach their former severity. Hereupon it was impossible to refrain from following what seemed to be the suggestion of nature herself, and to procure an artificial discharge as nearly as possible from the same parts where the spontaneous abscess had arisen. Accordingly, a large caustic issue was made on the inside of the left humerus, and a very copious discharge of pus maintained for a month. The symptoms, in the mean time, became more and more severe: the issue therefore was allowed to heal.

Still the absence of all pain during several weeks seemed irreconcileable with the existence of organic disease within the cranium. It was a duty, therefore, still to attempt something for her relief. She had already taken mercury, but its use was interrupted before it had been tried fairly: she now took mercury again, until she was profusely salivated; and the salivation was maintained during several weeks, but no benefit followed.

At length, so great was the patient's agony, that all attempts to cure were postponed to the purpose of obtaining for her that relief which opium could procure. Throughout the whole of the month of May, until her death on the 1st of June, opium and purgatives were the only medicines employed. Her bowels, during the whole course of her complaint, were obstinately constipated.

The character and magnitude of this poor creature's sufferings will be best conceived from a description of her condition during the last month of her existence.

The pain, without being confined to any one part of the head, belonged more especially to the occiput; it was constantly present, but varied in degree. Each twenty-four hours were about equally divided between a greater and a less degree of suffering. About two o'clock in the morning began the period of her greatest agony, which continued until about two in the afternoon. She was generally seen by Dr. Latham about mid-day, when she presented a picture of bodily suffering carried to its most extreme degree, and it was reported that she had been exactly in the same state for eight or ten hours. She lay on one side, generally on the left, with her countenance flushed, her eyes closed, and her eyebrows, eyelids, forehead, and temples, knitted and drawn together. Her head was immoveable from one position; it was sunk down towards the shoulders, and rigidly fixed there. Her hands were clenched. To stir any muscle of the body which could induce the slightest motion of the head, immediately augmented her agony. To unclose an eye, or to utter a single word in answer to questions put to her, was to aggravate her torture. On one occasion a gentleman, who came to see her, let an orange drop upon her pillow: immediately her agony was manifested by her seizing and tearing the pillow with her teeth.

As the evening advanced, she was able to allow the muscles of her head and face gradually to relax themselves. She opened her eyes, and could take a little liquid food. Towards nine o'clock she began to dose, and from this time until two in the morning she obtained some sleep by snatches; when the severity of her agony began again.

She died on the 1st of June. As the period of her dissolution approached, the intervals became shorter and shorter in which she experienced any mitigation of her pain, and during the last fortnight of her existence she was in one continual agony. Three days before she died, her left leg was paralysed, but she preserved her senses and intellect to the last.

*Sectio cadaveris.*—Upon dissection, the brain and its membranes were found a little more vascular than natural. There was some effusion beneath the arachnoid, and a considerable quantity (perhaps two ounces) of serum in the lateral ventricles, and the membrane lining them was very much thickened. No other morbid appearance presented itself in any part of the brain.

At the base of the cerebellum, growing from both lobes, but in a greater degree from the left, was a tumor, which descended beneath the dura mater into the spinal canal, and reached as low as the origin of the sixth pair of cervical nerves. This tumor had the appearance and consistence of fœtal brain. It grew out of the interior of the cerebellum, and seemed to form a part of it; the cerebellum in its neighbourhood being softened down to the same consistence with the tumor itself. It covered the posterior part and sides of the spinal marrow as far as it reached, and rested upon the origins of the nervus accessorius, and of all the cervical nerves as low as the sixth; but it did not involve any of them in its substance. A small portion of coagulated blood adhered to the lower extremity of the tumor.

Blood was extravasated in small quantities upon the pia mater of the spinal marrow, here and there throughout its whole course. No other disease was found in any part of the body.

